# Mucous membrane pemphigoid involving the upper respiratory tract: A case series and literature review

**DOI:** 10.1097/MD.0000000000044680

**Published:** 2025-09-26

**Authors:** Yuqi Wang, Xingli Zhou, Jie Yu, Yuhao Zou, Haiyang Wang, Wei Li, Jian Zou

**Affiliations:** a Department of Otolaryngology-Head & Neck Surgery, West China Hospital, Sichuan University, Chengdu, Sichuan Province, China; b Department of Dermatology & Venerology, West China Hospital, Sichuan University, Chengdu, Sichuan Province, China; c West China Clinical Medical College of Sichuan University, Chengdu, Sichuan Province, China.

**Keywords:** diagnosis, mucous membrane pemphigoid, prognosis, symptom, treatment, upper respiratory tract

## Abstract

**Rationale:**

We present a case series involving 4 patients diagnosed with upper respiratory tract mucous membrane pemphigoid (MMP) at a tertiary referral center, accompanied by a comprehensive literature review.

**Patient concerns:**

This study focuses on cases of MMP that presented with respiratory symptoms and were treated at the Department of Otolaryngology-Head and Neck Surgery, West China Hospital, from 2018 to 2023. In addition, it reviews cases of MMP affecting the upper respiratory tract that have been published on PubMed from 2013 to 2023.

**Diagnoses:**

Four patients were diagnosed with MMP affecting the upper respiratory tract.

**Interventions:**

One patient required a tracheotomy due to severe dyspnea, while the other 3 initially presented with symptoms affecting various regions of the upper respiratory tract. Diagnosis for 3 of the patients was confirmed through direct immunofluorescence of skin or mucosal samples, in conjunction with pathological examination, and they were subsequently treated with systemic glucocorticoids.

**Outcomes:**

Notably, the second patient exhibited favorable outcomes when glucocorticoids were combined with methotrexate. The third patient’s condition showed gradual improvement following the initial glucocorticoid treatment, and intravenous immunoglobulin and rituximab were later administered to alleviate symptoms. Following the tracheotomy, the fourth patient’s disease was effectively managed with regular corticosteroid treatment in the Dermatology Department.

**Lessons:**

Considering the prevalent symptoms associated with upper respiratory tract MMP and the severe repercussions of failing to intervene, early diagnosis and the timely administration of systemic corticosteroids are of paramount importance.

## 1. Introduction

Mucous membrane pemphigoid (MMP) is an immune-mediated blistering disorder that primarily affects the mucosal surfaces. Due to its characteristic tendency to form blisters that readily rupture, resulting in ulcerations and subsequent scarring, the condition was previously referred to as “scarring pemphigoid.”^[1]^ The annual incidence of MMP is estimated to be approximately 1 in 1000,000, with a higher prevalence observed in females and a typical age range of 60 to 80 years.^[2]^ MMP is classified as an autoimmune subepithelial blistering disease predominantly affecting mucous membranes, and it is characterized by the presence of autoantibodies targeting various autoantigens within basement membrane zone (BMZ) proteins, including collagen XVII (COL17). The pathogenesis of MMP is believed to be associated with multiple factors, including medications, injuries, and infections.^[3,4]^

It is important to note that the involvement of the upper respiratory tract (which includes the nasal, pharyngeal, and laryngeal regions) and the alimentary tract (comprising the upper sections: esophagus, stomach, and duodenum, as well as the lower sections: jejunum, ileum, and large intestine) is exceedingly rare, with reported prevalence rates of 20 to 40% in the nasal region, 20 to 40% in the pharynx, 5 to 10% in the larynx, and 5 to 15% in the esophagus.^[5–7]^ The objective of this article is to summarize the differences in symptoms, diagnosis, treatment, and prognosis of MMP affecting the upper respiratory tract and the alimentary tract.

## 2. Materials and methods

**Figure 1.**
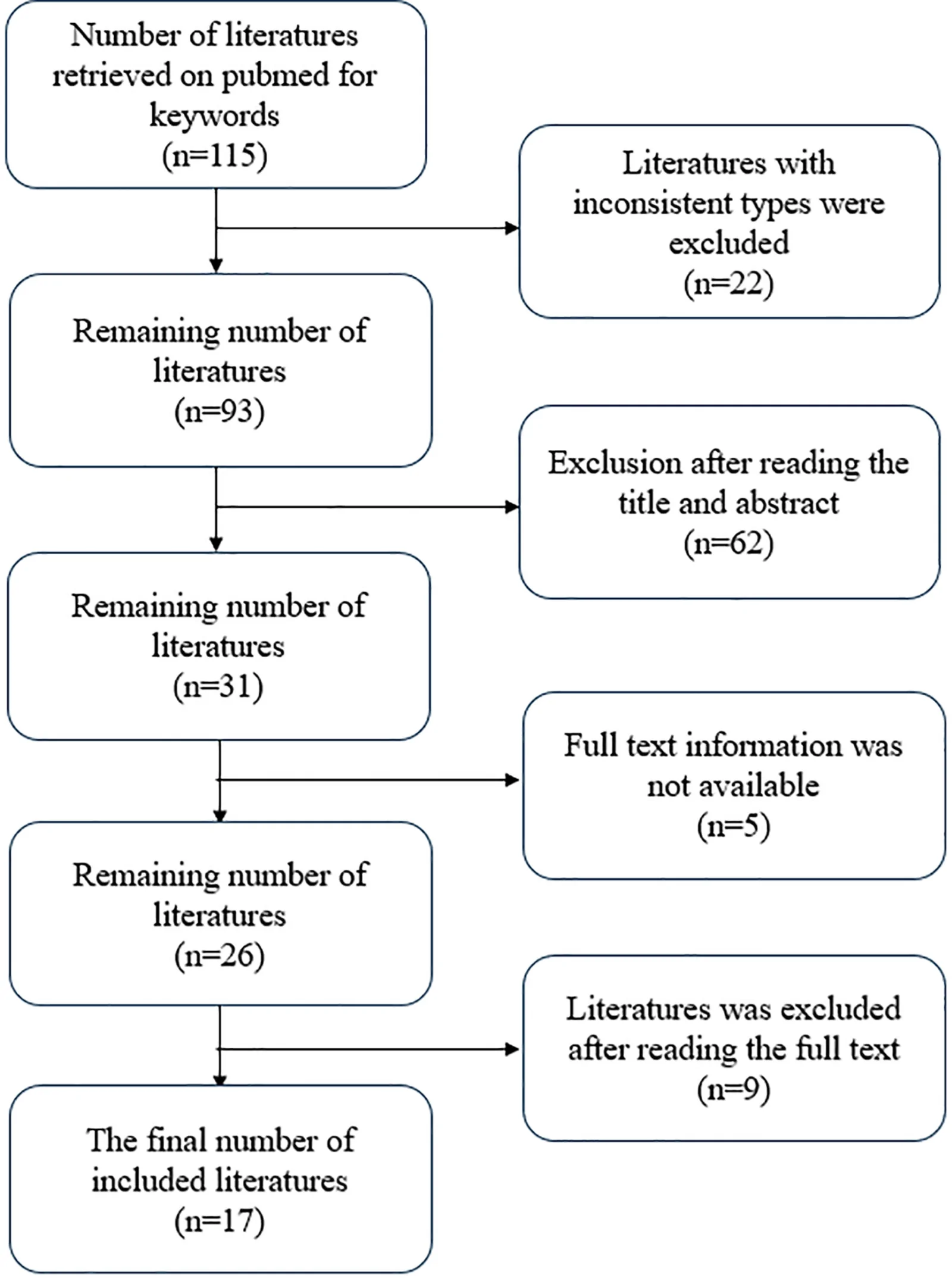
Literature search flow chart.

This article presents a retrospective case record review of patients diagnosed with MMP at West China Hospital from 2018 to 2023. The study included patients who exhibited upper respiratory tract manifestations (specifically in the nose, pharynx, and larynx) during the course of their illness and who received regular treatment at the hospital. Patients with incomplete information were excluded from the study. Data were meticulously collected from patient records by a team comprising one otolaryngologist and one dermatologist. The collected data encompassed demographic information, clinical presentations, immunological findings, histological findings, endoscopic reports, pharmacological and surgical management, follow-up processes, and clinical prognostic evaluations. Additionally, the data were analyzed by another otolaryngologist and dermatologist. Ethical approval for the study was obtained from the Biomedical Ethics Committee of West China Hospital, Sichuan University 2017 (214). The patients in this manuscript have given written informed consent to publication of their case details. Furthermore, this article includes a literature review summarizing all publicly available full-text cases of MMP involving the upper respiratory tract published on PubMed between 2013 and 2023. The literature search utilized keywords (“nose” or “nasal” or “nasopharynx” or “oropharynx” or “laryngopharynx” or “larynx” or “pharynx” or “throat”) and (“mucous membrane pemphigoid” or “scarring pemphigoid” or “benign mucosal pemphigoid” or “mucosal pemphigoid”). A total of 113 articles were retrieved; however, exclusion criteria included inconsistencies in literature type, content discrepancies, and a lack of information regarding symptoms, diagnosis, and treatment. The inclusion criteria were limited to case reports and case series of MMP with upper respiratory symptoms. Ultimately, 17 articles met the inclusion criteria. The flowchart detailing the literature retrieval process is presented in Figure 1.

## 3. Results

### 3.1. Case 1

**Figure 2.**
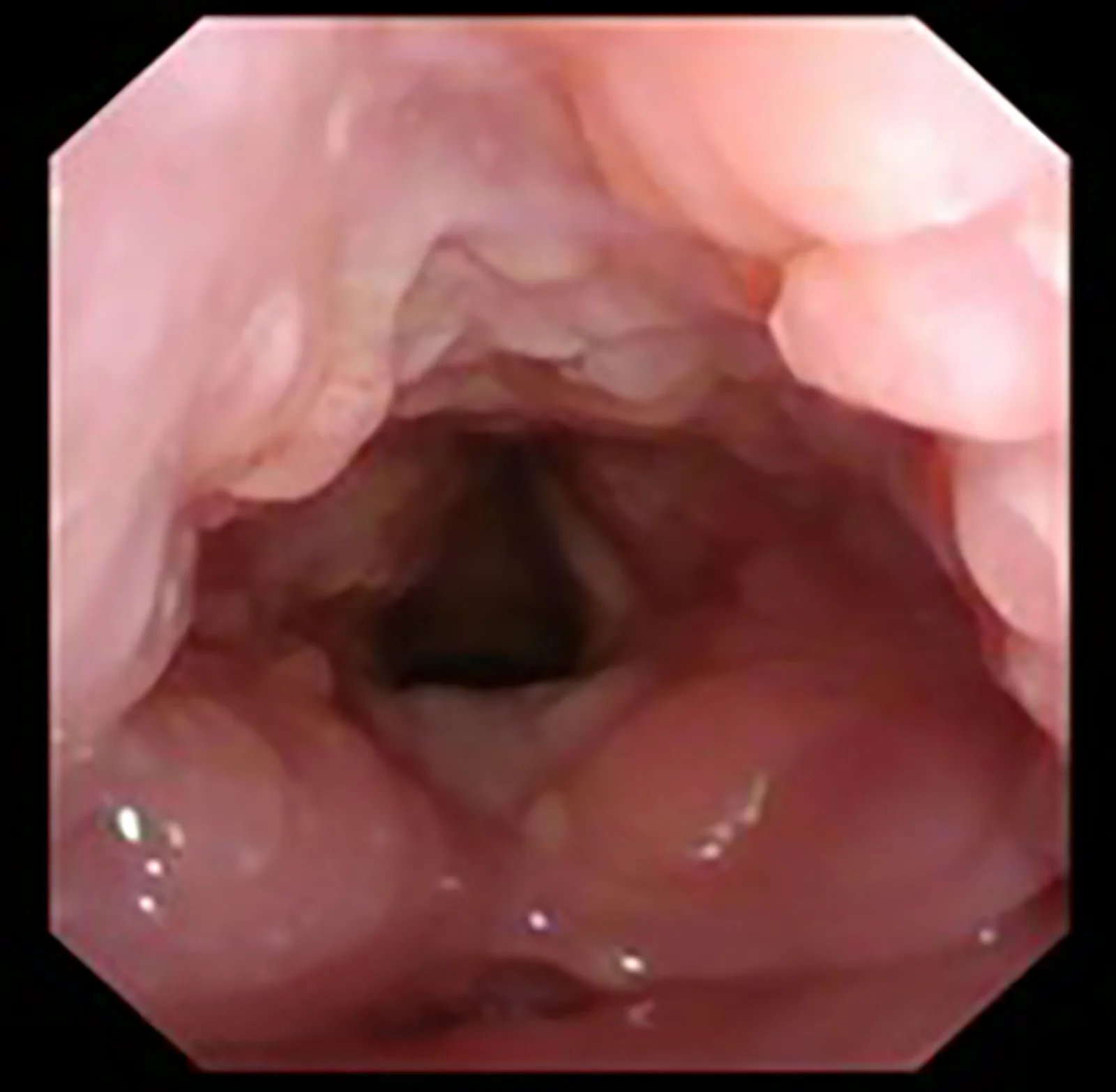
Case 1 revealed considerable nodular hyperplasia in the larynx by fiberoptic laryngoscopy.

**Figure 3.**
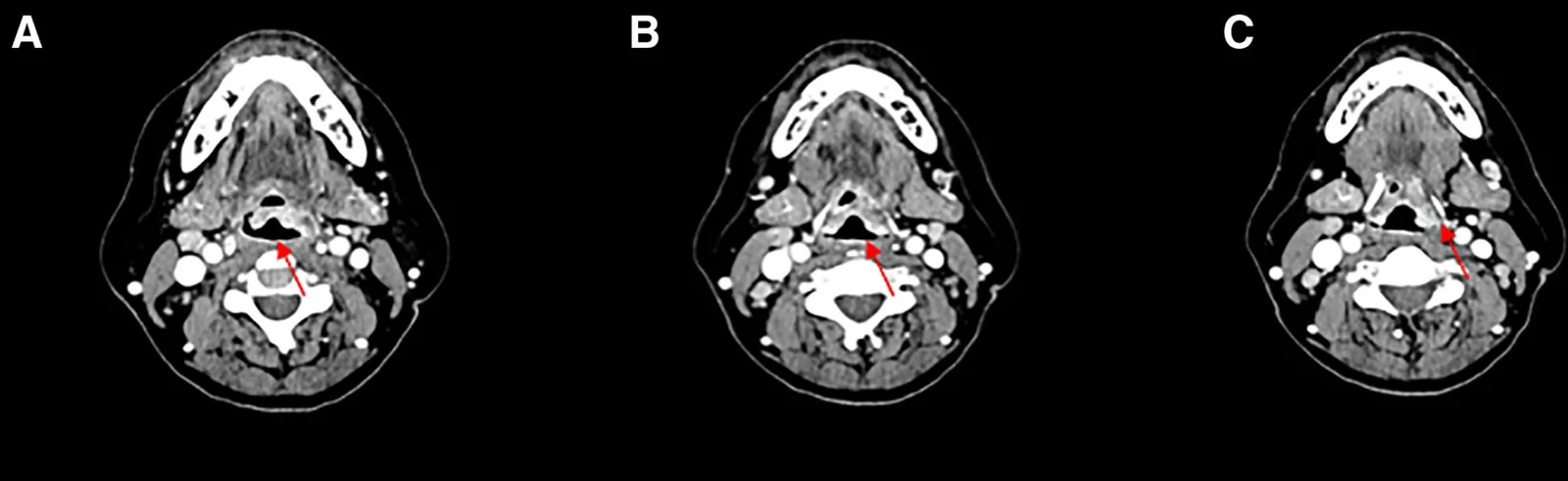
Computed tomography imaging of case 1. (A–C) Computed tomography images of 3 different levels of the pyriform fossa. Thickening and enhancement of the left epiglottic fold, with concomitant narrowing of the left anterior epiglottic space and the pyriform fossa.

**Figure 4.**
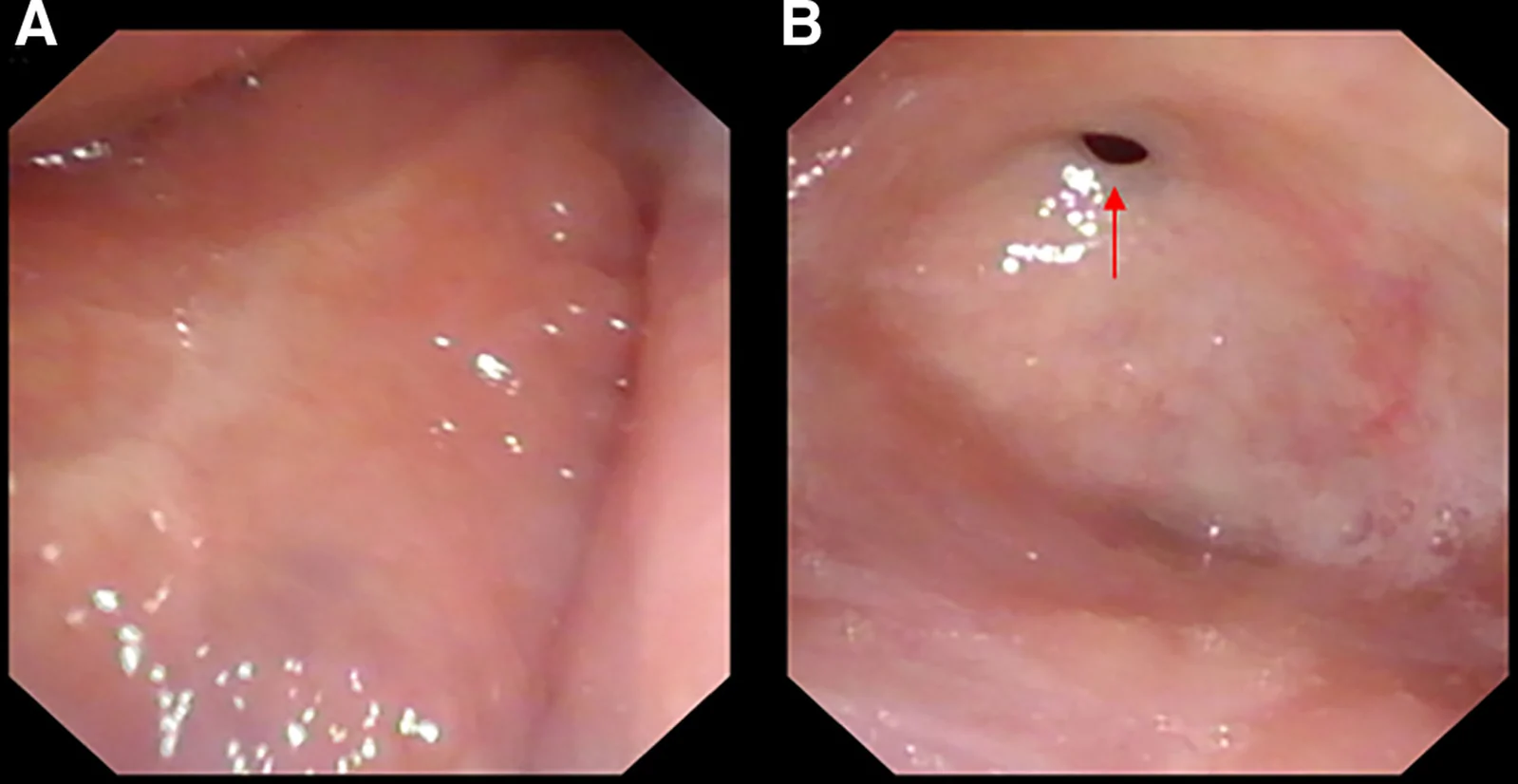
Case 1 detected localized scar-like alterations in the nasopharyngeal mucosa (A) and adhesions at the soft palate level, leaving only a small fissure (B) by fiberoptic laryngoscopy.

**Figure 5.**
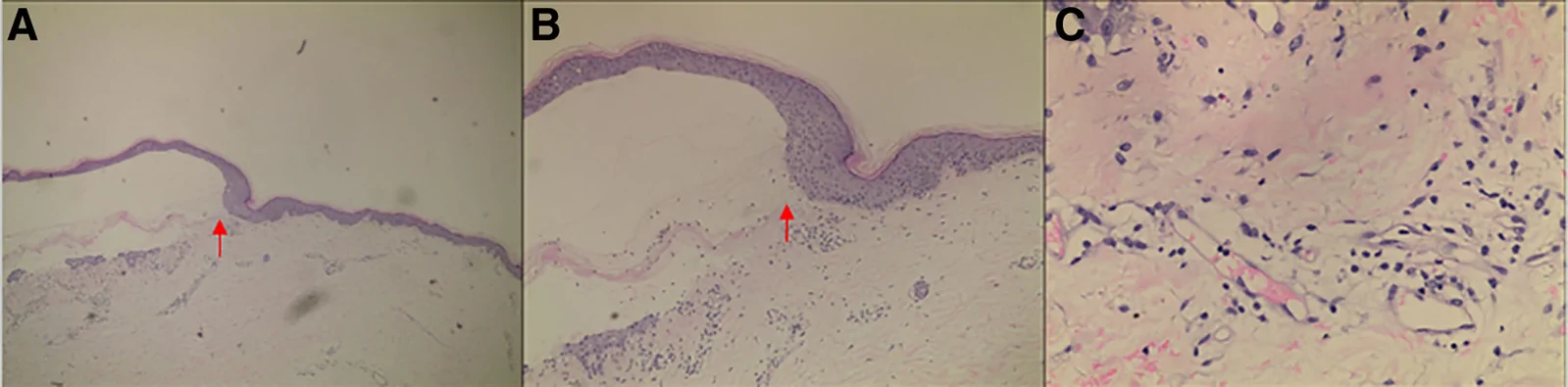
Case 1 found subepidermal splitting and infiltration by mixed inflammatory cells (A) 40×, (B) 100×, (C) 400× by pathological biopsy.

**Figure 6.**
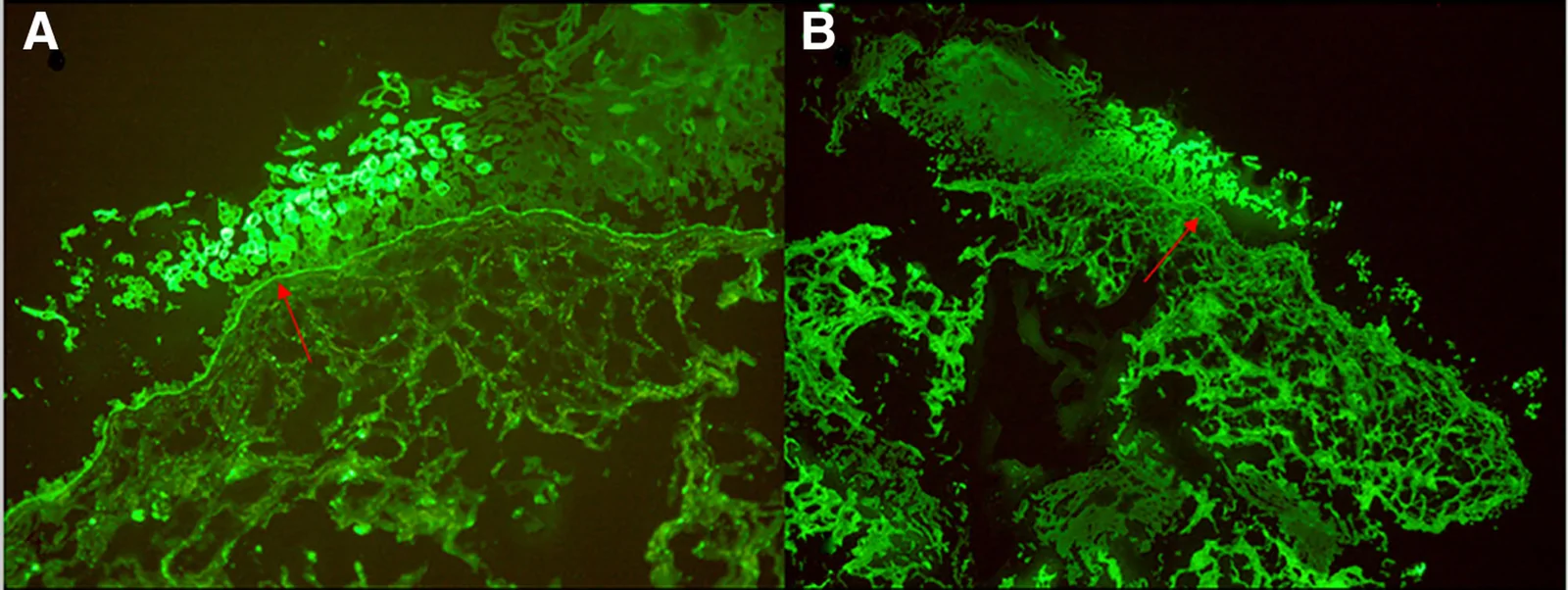
Case 1 found C3 (200× electron microscope, A) and IgG (100× electron microscope, B) were deposited linearly along the basement membrane by DIF.

A 58-year-old female patient presented to our otolaryngology department with a complaint of a foreign body sensation in her pharynx, accompanied by a persistent cough lasting 2 months. The patient had no significant past medical history. Initial treatment for suspected pharyngeal reflux disease involved the administration of Vonoprazan Fumarate Tablets at a dosage of 10 mg once daily; however, this intervention did not alleviate her symptoms after 3 days. Subsequent fiberoptic laryngoscopy revealed significant nodular hyperplasia in the larynx (Fig. 2). A pathological biopsy of the nodular hyperplasia indicated squamous epithelial hyperplasia with minor inflammatory exudates. Computed tomography (CT) imaging demonstrated pronounced thickening and enhancement of the left epiglottic fold, along with concomitant narrowing of the left anterior epiglottic space and the pyriform fossa (Fig. 3A–C). Over the course of 3 months, the patient’s condition progressed to include a sore throat, a persistent sensation of a foreign body in the pharynx, and dyspnea. A follow-up fiberoptic laryngoscopy revealed localized scar-like changes in the nasopharyngeal mucosa and adhesions at the level of the soft palate, resulting in a small fissure (Fig. 4A and B). The mucosa of the right pyriform fossa appeared smooth, in contrast to the left side, which was not visible. Irregular elevations were observed on the lingual and laryngeal surfaces of the epiglottis and both ventricular bands; however, the vocal cords remained unaffected, exhibiting partial smoothness with normal motility and closure. In the following month, the patient’s condition continued to evolve, leading to the development of oral ulcers and subsequent conjunctivitis. An interdisciplinary consultation suggested a high likelihood that the patient’s condition resembled MMP. A biopsy taken from the oral mucosa, approximately 1 to 2 cm around the ulcer, revealed subepithelial splitting and infiltration by mixed inflammatory cells in the submucosa (Fig. 5A–C). Direct immunofluorescence (DIF) analysis of the mucosa surrounding the lesions confirmed linear deposition of IgG and C3 at the epidermal-dermal junction (Fig. 6A and B). The patient was treated with oral methylprednisolone tablets at a dosage of 0.4 mg/kg/day in conjunction with methotrexate at a dosage of 10 to 20 mg per week for 3 months, which effectively controlled her condition. Follow-up fiberoptic laryngoscopy indicated resolution of the lesions in the larynx. Methotrexate was subsequently discontinued. The patient then reduced the dosage by 50 mg every 2 weeks until reaching 40 mg/day, followed by a reduction of 5 mg every month until reaching 20 mg/day, and subsequently by 2.5 mg every 3 months when maintaining a dosage of 10 mg/day. After a follow-up period of 6 months, there was no evidence of recurrence.

### 3.2. Case 2

**Figure 7.**
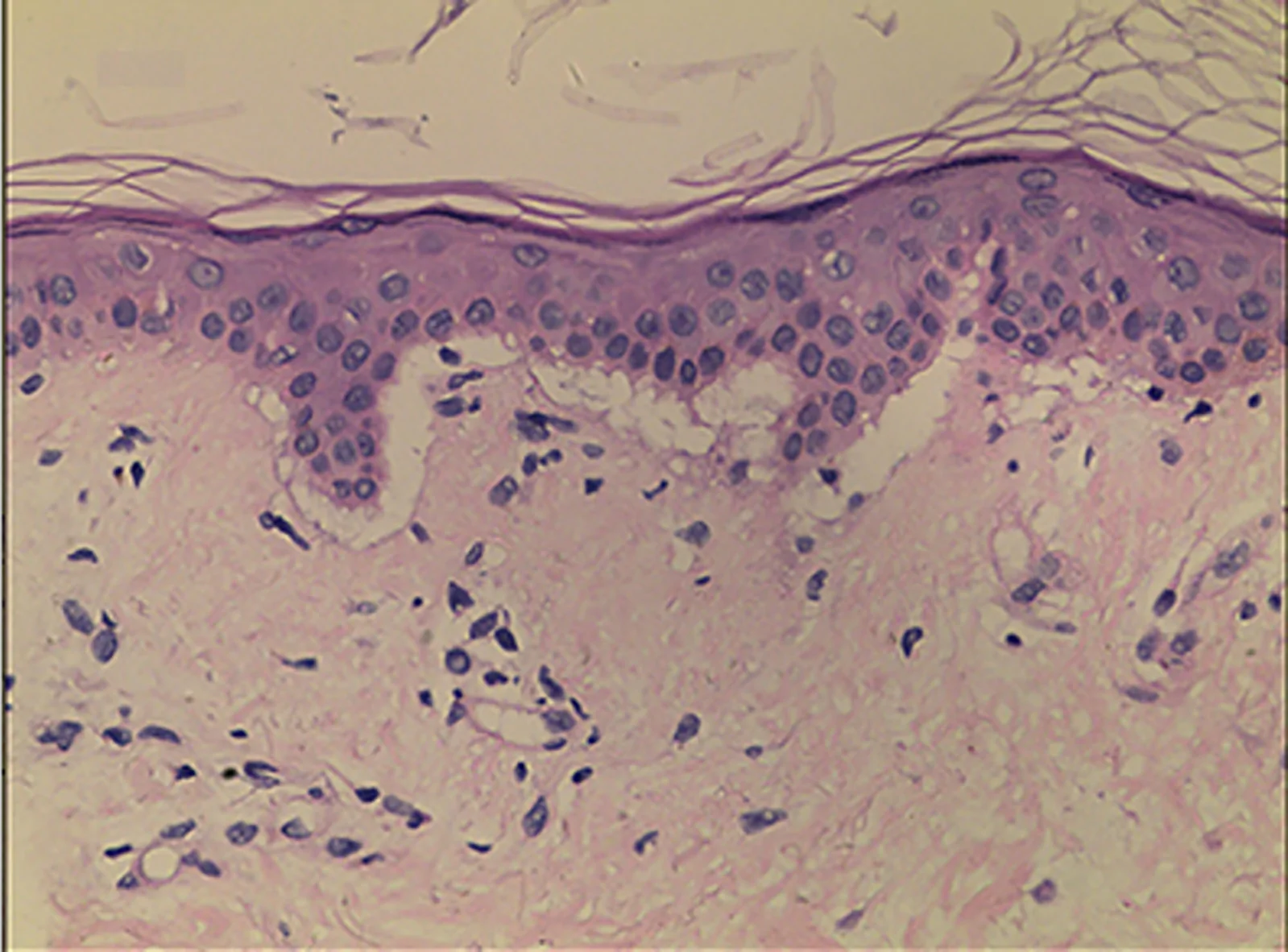
Case 2 revealed the presence of subepidermal blisters with a large lymphocytic infiltrate and a small eosinophilic infiltrate (400×) by skin pathological examination.

**Figure 8.**
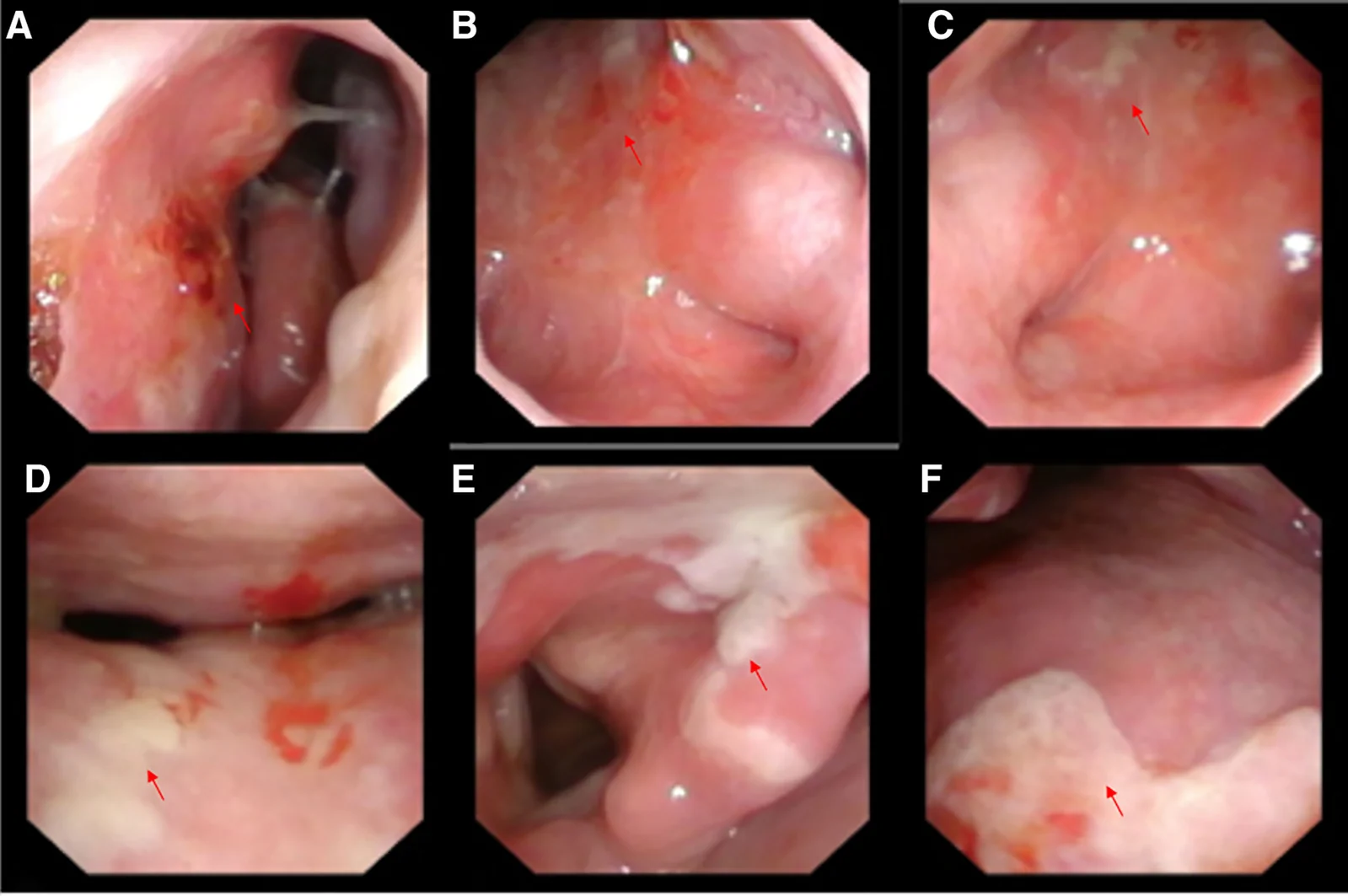
Case 2 found bilateral nasal cavities filled with mucus, the mucosa of the nasal septum showed erosions (A), and the posterior wall of the nasopharynx showed pseudomembrane formation and bloody secretions (B and C). Large ulcers were seen in the epiglottis of the larynx, affecting the right arytenoid region (D–F) by electronic nasopharyngoscopy.

**Figure 9.**
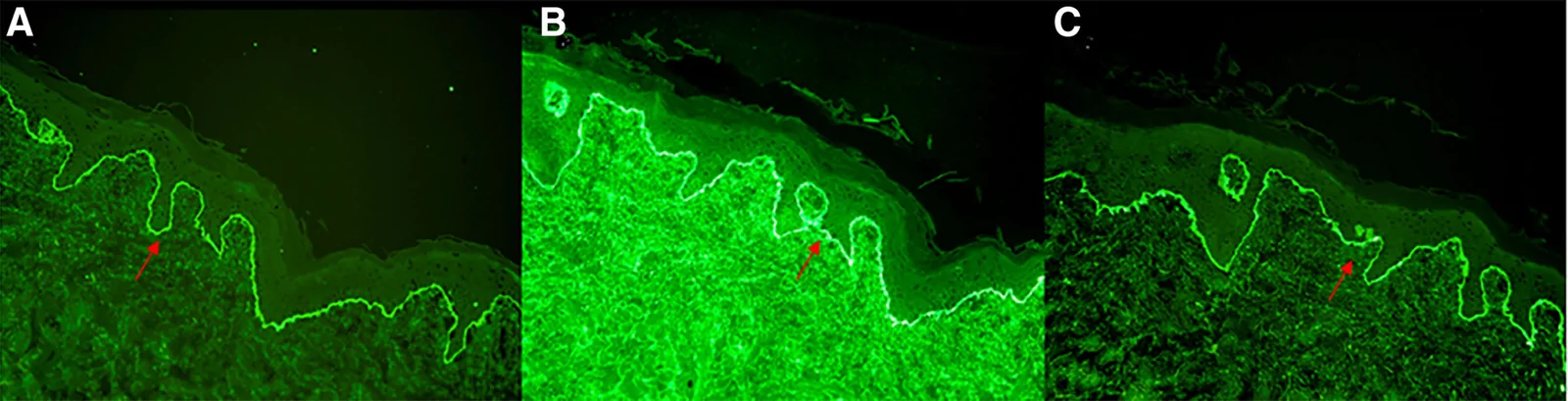
Case 2 found C3 (200× electron microscope, A), IgG (200× electron microscope, B) and IgM (200× electron microscope, C) were deposited linearly along the basement membrane by DIF.

**Figure 10.**
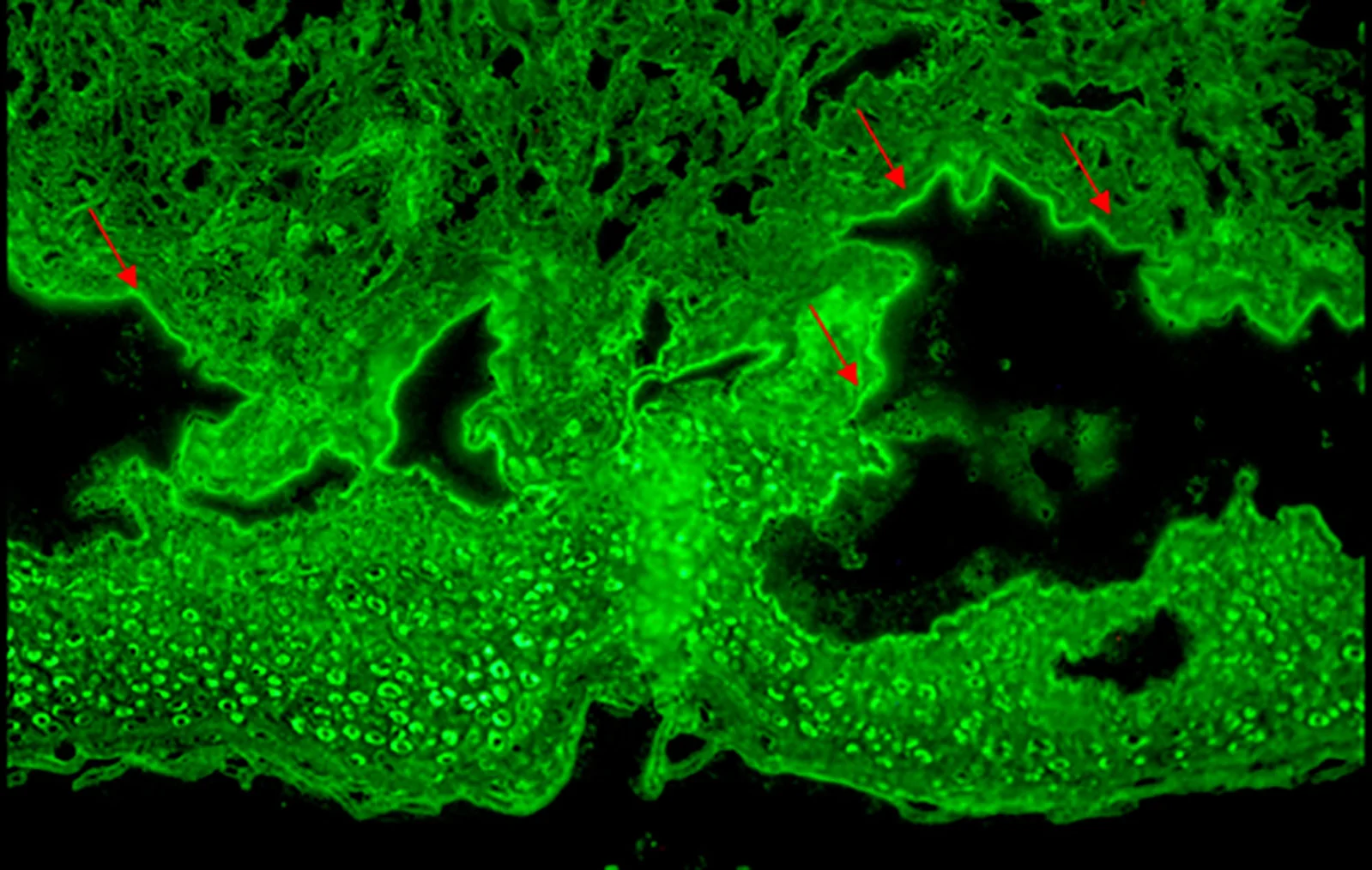
Case 2 found IIF-SSS was positive by indirect immunofluorescence. IIF-SSS = indirect immunofluorescence on salt-split skin.

The second patient was a 66-year-old male who initially presented to a local dental hospital with ulcers of the gingiva and bilateral buccal mucosa that had persisted for one month. Despite treatment with dexamethasone and lidocaine patches, the lesions did not resolve. Over the subsequent 6 months, the patient developed a series of skin ulcers, sore throat, dysphagia, and scalp blisters, leading to a referral to our hospital. Comprehensive immunological tests were conducted at our facility. Routine tests for MMP, including anti-bridging protein 1, anti-bridging protein 3, and anti-BP-180, yielded negative results. Additionally, other immunological assessments, such as ultrasensitive C-reactive protein and standard hematological tests, were within normal limits. A pathological examination of a 1 to 2 cm blister on the thigh revealed subepidermal blisters characterized by a significant lymphocytic infiltrate and a minor eosinophilic infiltrate in the dermis (Fig. 7A–C). For further diagnostic clarification, fiberoptic laryngoscopy was performed, and samples were obtained from the margins of the ulcers located in the right arytenoid region. The examination revealed bilateral nasal cavities filled with mucus, erosions on the mucosa of the nasal septum, and pseudomembrane formation with bloody secretions on the posterior wall of the nasopharynx. Large ulcers were observed on the epiglottis of the larynx, particularly affecting the right arytenoid region; however, no significant lesions were noted on the vocal cords bilaterally, and both vocal cord closure and vocal fold function remained normal (Fig. 8A–F). DIF testing of the larynx demonstrated linear deposits of IgG, C3, and IgM along the basement membrane between the epidermis and dermis (Fig. 9A–C). Indirect immunofluorescence on salt-split skin (IIF-SSS) was positive on both the epidermal and dermal sides (Fig. 10). Based on the clinical manifestations and immunofluorescence results, the patient was ultimately diagnosed with MMP. The patient subsequently commenced standard treatment for MMP. Due to the severity of this patient’s condition compared to the previous case, an oral regimen of methylprednisolone at a dosage of 1.0 mg/kg/day was initiated, followed by the addition of methotrexate at 10 to 20 mg/week and tofacitinib citrate at 11 mg/day. The administration of tofacitinib citrate was halted after 2 weeks due to the development of low back pain. Following one month of treatment with methylprednisolone in conjunction with methotrexate, the patient reported significant relief. Methotrexate was then discontinued, and maintenance therapy with methylprednisolone was continued. Subsequent fiberoptic laryngoscopy indicated resolution of the laryngeal lesions. The patient began a gradual reduction of the methylprednisolone dosage, with a 10% decrease biweekly, transitioning from 60 mg/day to 20 to 40 mg/day over the course of several months, ultimately reaching a long-term maintenance dose of 10 mg/day. Although the patient continued to experience occasional small skin blisters, there was no further progression of the disease. After one year of follow-up, the patient exhibited no signs of recurrence.

### 3.3. Case 3

**Figure 11.**
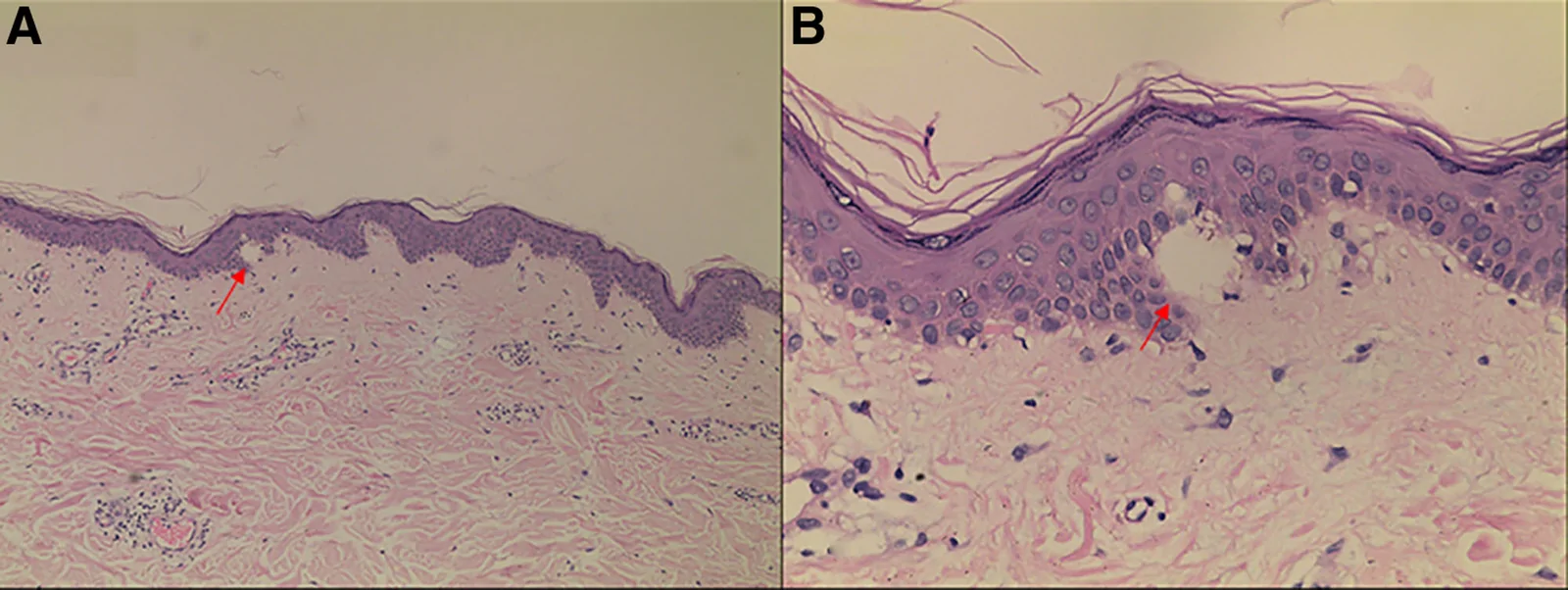
Case 3 indicated subepidermal blisters by pathological examination. Image A was captured at 40x magnification, while image B was obtained under 100x magnification.

**Figure 12.**
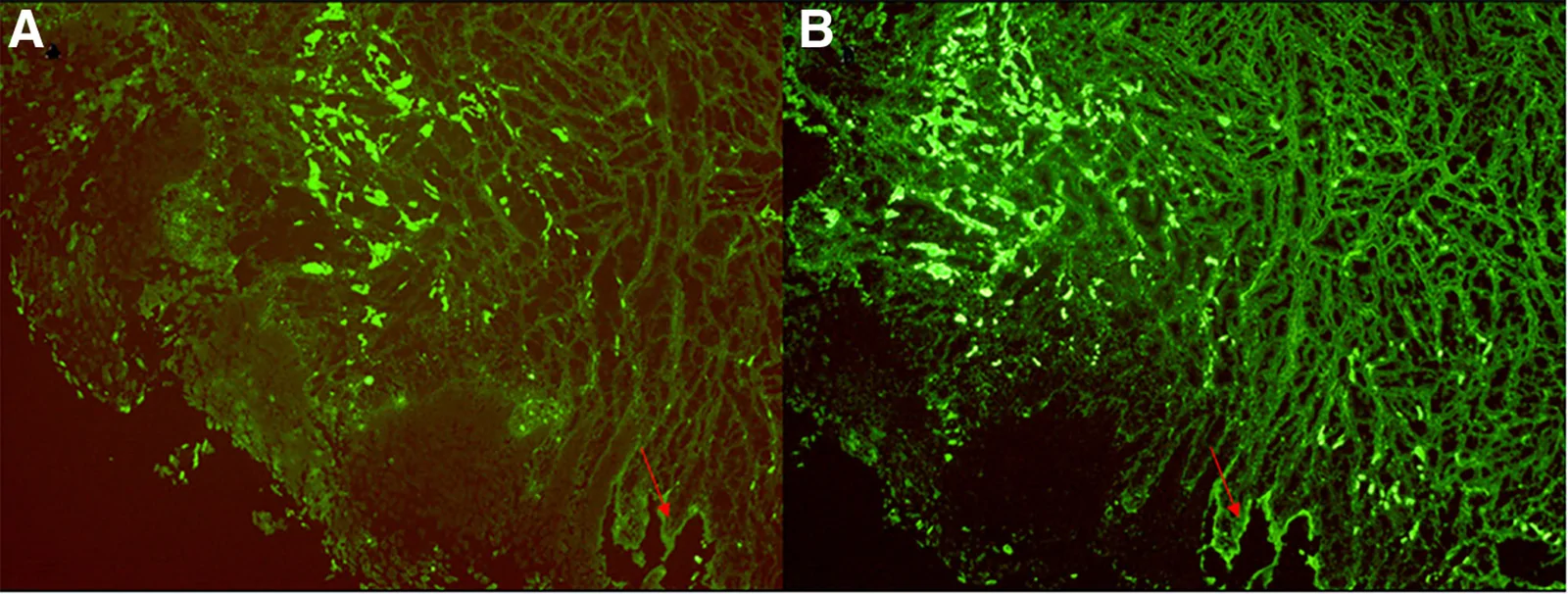
Case 3 found IgA (100× electron microscope, A) and IgM (100× electron microscope, B) were deposited linearly along the basement membrane by DIF.

The third patient in this case study is a 50-year-old female who presented to our otorhinolaryngology outpatient clinic with a 2-month history of unexplained rhinorrhea and intranasal adhesions. The patient had no significant medical history, including prior occurrences of nasal tumors, nasal inflammation, or nasal septum deviation. Routine coagulation tests conducted were within normal limits. Given the ambiguous etiology of her symptoms, a rhinotomy was performed to address the nasal adhesions. During the surgical intervention, extensive erosions and ulcers were identified within the nasal cavity. Postoperatively, the patient continued to experience nasal obstruction and rhinorrhea, which showed no signs of improvement. One month following the surgery, adhesions reformed, and the patient developed refractory oral ulcers. Her condition deteriorated 3 months post-surgery, leading to the emergence of skin blisters and ulcers, accompanied by a foreign body sensation in the pharynx. Consequently, she sought evaluation at a dermatology clinic, where biopsies were obtained from the margins of the blisters located on her lower extremities and within her oral cavity. Dynamic laryngoscopy revealed significant purulent secretions and crusting in both the bilateral nasal cavity and nasopharynx, as well as stenosis in the nasopharynx (see Supplemental Video 1, which demonstrates the degrees of the erosion, bleeding and stenosis of the nasal cavity). Pathological examination of the oral mucosa demonstrated the presence of subepidermal blisters, a marked infiltration of lymphocytes surrounding small blood vessels in the superficial dermis, along with individual eosinophils and neutrophils (Fig. 11A and B). DIF of oral mucosa indicated the presence of linear IgM and IgA along the BMZ (Fig. 12A and B). Due to the severity of the patient’s symptoms, we initiated treatment with oral methylprednisolone at a dosage of 0.5 mg/kg/day. After one week, as there was no significant improvement, we escalated the treatment to methotrexate at a dosage of 10 mg/week for home administration. However, the patient’s condition remained poorly controlled, prompting the addition of immunoglobulin therapy at a dosage of 400 mg/kg/day. This intervention successfully stabilized the patient’s condition. Following one week of immunoglobulin treatment, the patient achieved remission and was subsequently maintained on oral methylprednisolone at a reduced dosage of 0.3 mg/kg/day. There were no recurrences of nasal stenosis or epistaxis observed during subsequent nasal endoscopy. After 4 months of follow-up, the patient was managed with topical halometasone cream alone.

### 3.4. Case 4

**Figure 13.**
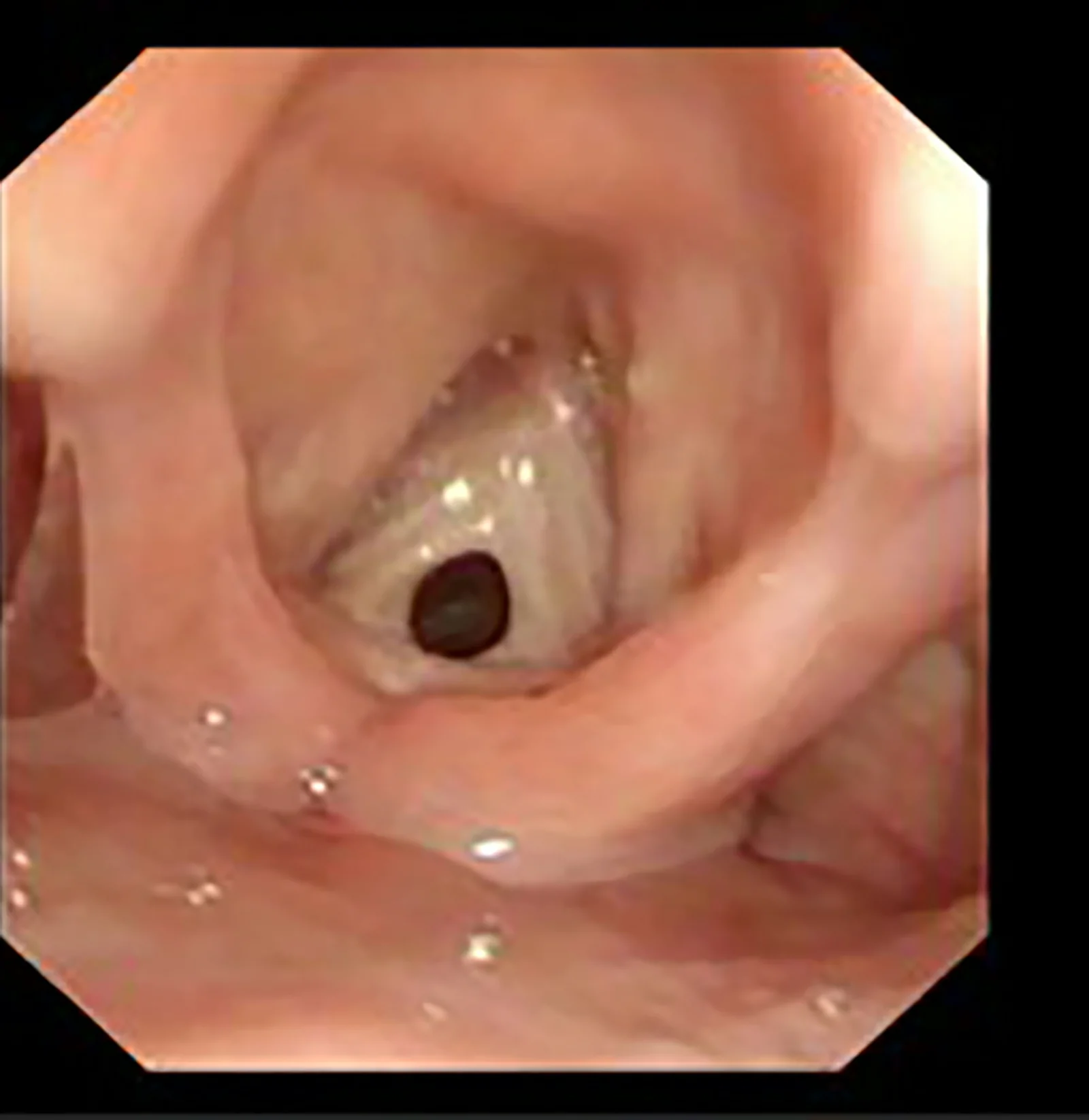
Case 4 was found to have annular glottic stenosis by electronic nasopharyngoscopy after tracheotomy.

The fourth patient in this case study was an 80-year-old female who presented to our otolaryngology clinic with severe dyspnea and laryngeal stridor. She had previously undergone a tracheotomy 6 months prior and sought evaluation for the potential removal of the cervical endotracheal tube. Upon conducting a fiberoptic laryngoscopy, we noted that the left nasal alae were collapsed and adhered, allowing access to the left nasal cavity via endoscopy. A significant amount of purulent secretions was observed in the right posterior nostril. Examination of the bilateral vocal cords revealed annular contraction with smooth surfaces, and the glottis appeared relatively narrow (Fig. 13). The tracheostomy opening was unobstructed, and the tracheal cannula remained correctly positioned. Based on our clinical experience, we hypothesized that the patient may be suffering from a condition related to pemphigus or pemphigoid. Consequently, she was referred to the dermatology department for further evaluation. The examination of MMP, pathological and immunofluorescence results, confirmed the diagnosis of MMP. The dermatology department recommended treatment with oral methylprednisolone at a dosage of 1.5 mg/kg/day. Following 20 days of treatment, the patient’s condition showed significant improvement, and subsequent fiberoptic laryngoscopy indicated that the laryngeal lesions had not progressed. The dosage of methylprednisolone was subsequently reduced by 10% every 2 weeks. When the dosage reached between 40 to 60 mg/day, it was decreased by 5 mg every 2 weeks. For dosages between 20 to 40 mg/day, the reduction was adjusted to 5 mg per month. Once the dosage reached 20 mg/day, it was further reduced by 2.5 mg every 3 months, ultimately tapering to a long-term maintenance dose of 0.2 mg/kg/day. The patient was evaluated for decannulation 3 months after the initiation of maintenance therapy. Notably, no episodes of dyspnea were reported during the 6-month follow-up period.

Table 1 summarizes key demographic and clinical data of the 4 patients.

**Table 1:** Patients demographic and clinical data.

Age (yr)	Gender	Initial symptom	Upper respiratory tract involvement	Other areas	Degree of disease	Pathological biopsy	DIF/IIF/ELISA/IB	Treatment	Prognosis and outcome	Follow-up treatment
Case 1 (58)	Female	Foreign body sensation in pharynx, Cough	Nasopharynx, Larynx (Lingual and laryngeal surfaces of epiglottis)	Ocular, Oral	Ulcer, Erosion	Dermal division, infiltration of mixed inflammatory cells	DIF: linear deposition of IgG/C3 anti-BMZ	Oral glucocorticoids + methotrexate	Relapse	A small dose of glucocorticoid
Case 2 (66)	Male	Oral ulcer	Nasal, Nasopharynx, Larynx (The laryngeal surface of epiglottis), Arytenoidea	Skin	Pseudomembranous, Ulcer, Bloody discharge	Subepidermal blisters with a large number of lymphocytes accompanied by a small number of eosinophils	DIF: linear deposition of IgG/C3/IgM anti-BMZ, IIF: salt-split skin(+)	Methylprednisolone + Methotrexate + Tofaciib citrate	Relapse	A small dose of glucocorticoid
Case 3 (50)	Female	Rhinorrhagia, Nasal adhesions	Nasal, Nasopharynx, Larynx	Skin	Ulcer, Erosion	Subepidermal blisters, a large number of lymphocytes and individual eosinophils and neutrophils infiltrated around small vessels in the superficial dermis	DIF: linear deposition of IgM/IgA anti-BMZ	Glucocorticoid + IVIG + Rituximab	Relapse	/
Case 4 (80)	Female	After tracheotomy	Pharynx, Larynx	/	Scar	/	/	Tracheotomy	/	/

BMZ = basement membrane zone, DIF = direct immunofluorescence, ELISA = enzyme linked immunosorbent assay, IB = immunoblotting, IIF = indirect immunofluorescence, IVIG = intravenous immunoglobulin.

### 3.5. Literature review

From 2013 to 2023, a total of 17 cases of MMP involving the upper respiratory tract were documented.^[3,6,8–22]^ The study conducted by Wells et al reported 2 cases, while the remaining literature consisted primarily of individual case reports. The mean age of the patients was 63.94 ± 14.35 years, with a range of 41 to 85 years. Among the patients, 7 were male (41.18% of the total) and 10 were female (58.82% of the total). Laryngeal involvement was observed in 15 patients, pharyngeal involvement in 8, and nasal involvement in another 8. Additional sites of involvement included the eyes (10 cases), skin (6 cases), and mouth (6 cases), as well as the esophagus, trachea, and bronchi. Common presenting symptoms included dysphagia, hoarseness, laryngeal stridor, chronic sinusitis, sore throat, cough, dyspnea, and rhinorrhea. Fiberoptic laryngoscopy revealed erythema, swelling, blisters, ulcers, pseudomembrane formation, and scar formation, with the severity of these findings varying according to the extent of the lesions. The pathological manifestations were predominantly characterized by mixed inflammatory cell infiltration. Linear deposits of IgG and C3 were identified along the basement membrane in 7 patients, while IgG, C3, and IgA were detected in 2 additional patients, and IgA alone in 1 patient. IIF-SSS was positive in 2 patients. Anti-Laminin (LM)-332 was identified in 4 patients, anti-BP-180 in 1 patient, and anti-α4β6 in 1 patient, as determined by enzyme-linked immunosorbent assay (ELISA)/immunoblotting (IB). Treatment typically involves the administration of systemic corticosteroids in conjunction with cyclophosphamide, azathioprine, ampicillin, and mycophenolic acid. In cases of relapse, rituximab and immunoglobulin are frequently employed. In instances where symptoms are severe, surgical interventions such as tracheotomy, CO2 laser surgery, and supraglottic balloon dilation may be indicated.

Table 2 summarizes key demographic and clinical data of the 17 literatures.

**Table 2:** Literature review of previous cases involved upper respiratory tract.

Author, year	Age (yr)	Gender	Initial symptom	Upper respiratory tract involvement	Other areas	Degree of disease	Pathological biopsy	DIF/IIF/ELISA/IB	Treatment	Prognosis and outcome	Follow-up treatment
Murono, S. et al,^[8]^ 2013	69	Male	Difficulty swallowing	Larynx (pyriform sinuses, epiglottis, postcricoid hypopharynx)	Skin	Scar	/	/	Surgery	/	/
Kato, K. et al,^[9]^ 2014	76	Female	Pain in the mouth, Hoarseness	Pharynx, Larynx	Ocular, Skin	Ulcer, Erosion	Subepithelial cleft, Lymphocytic infiltration	DIF: linear deposition of IgG/C3 anti-BMZ IIF: 1: 40 circulating IgG anti-BMZ bound to the dermal side, ELISA/IB: Anti-LM-332	Systemic prednisolone 50 mg daily and cyclophosphamide pulse (500 mg every 4 wk)	Relapse after remission, Final death	Plasma replacement
Schempf, U. et al,^[10]^ 2014	41	Female	Dysphagia, stridor	Larynx (vocal cords)	Esophagus	Scar	Inflammation	/	Dilation therapy	/	/
Brophy, J. et al,^[11]^ 2015	43	Female	Chronic sinusitis, Extensive nasal scarring, stridor	Nasal, Larynx	Ocular	Scar	/	DIF: linear deposition of IgG/C3 anti-BMZ	Systemic corticosteroids + IVIG	Partial remission (Conjunctiva)	/
Lambiel, S. et al,^[6]^ 2017	73	Female	Oral burning Sensation, Sore throat, Cough, Hoarseness, Dyspnea	Nasal (middle turbinate, nasopharynx), Pharynx, Larynx (supraglottic, posterior hypopharyngeal wall)	Skin, Oral	Scar	Subepithelial cleft, Eosinophilic infiltration	DIF: linear deposition of IgG/C3 anti-BMZ ELISA/IB: anti-BP180, anti-LM332	Intravenous dexamethasone 4 mg tid, 3 d followed oral prednisone (30 mg/d) + dapsone (50 mg/d during the 1st wk; 75 mg/d during the 2nd wk; and 100 mg/d thereafter)	Partial remission (12 mo) followed by recurrence (Larynx)	Rituximab + a transitory increase of prednisone and dapsone doses (good clinical response)
Mendoza, A. et al,^[12]^ 2017	66	Female	Dyspnea	Larynx (supraglottic)	Ocular, Oral	Blister	Subepithelial blister, Mixed inflammatory cells (lymphocytes, neutrophils, eosinophils)	DIF: linear deposition of IgG/C3 anti-BMZ	Corticosteroid + cyclophosphamide, Tracheostomy	Died from a myocardial infarction	/
Rovas, G. et al,^[13]^ 2019	44	Male	Dyspnea, Cough, Hoarseness, Stridor	Nasal, Larynx (glottis, anterior union)	Oral, Ocular	Blister, Erosion	/	DIF: linear deposition of IgG/C3 anti-BMZ ELISA/IB: anti-LM-332	CO2 laser surgery, prednisolone + mycophenolate mofetil (MMF)	Complete clinical and serological response	/
Brake, D. A. et al,^[14]^ 2020	80	Female	Pharyngeal discomfort, Noisy breathing	Nasal, Larynx (supraglottic, epiglottis)	Ocular	Swell, Ulcer, Scar	/	/	Steroids + Mycophenolic acid	Dysphagia relief but dyspnea not	Rituximab 1000 mg (Infusions every 2 wk) (Complete remission, 10 mg prednisone twice daily)
Elmas, L. et al,^[15]^ 2020	68	Female	Dyspnea	Pharynx(hypopharynx), Larynx	/	Erythema, Ulcer, Pseudomembranous	Subepithelial clefting with fibrin, exudate, mixed inflammatory cells	DIF: linear deposition of IgA anti-BMZ	Tracheostomy, 80 mg/d oral prednisolone	Partial remission	Prednisolone + dapsone (Significant remission)
Law, S. et al,^[16]^ 2020	72	Female	Throat discomfort, Cough	Larynx (epiglottis), Pharynx (posterior pharyngeal wall)	/	Erythema, Ulcer	/	/	Steroid + Intralesional triamcinolone injections	Significant remission	/
Watters, C. T. M. et al, ^[17]^ 2020	45	Male	Sore throat, Dysphonia	Nasal, Larynx (glottis)	Ocular, Oral, Skin	Crusting, Inflammation, Ulcer	/	/	Corticosteroids + Dapsone + Mycophenolate	Significant remission	/
Lannan, M. et al,^[18]^ 2022	55	Male	Cough, Progressive shortness of breath	Pharynx (oropharynx), Larynx	Ocular, Tracheobronchial	/	/	/	Prednisolone + Mycophenolate	Partial remission	Cyclophosphamide, rituximab, IVIG (Significant remission)
Quattri, E. et al,^[19]^ 2022	74	Female	Not obvious	Nasal, Pharynx (oropharynx)	Ocular, Oral, Skin	/	Subepidermal blisters, Lymphocytic infiltrates, rare eosinophils	DIF: linear deposition of IgG/C3 anti-BMZ IIF: linear IgG deposition along the blister floor ELISA/IB:Anti-LM-332	Triamcinolone acetonide 0.1% in orabase once daily for 30 d and oral prednisone 0.75 mg/kg/d	Relapse after remission (pharyngolaryngeal synechiae)	Tracheostomy
Wells, H. et al,^[20]^ 2022	52	Female	Oral blisters, Nosebleeds, Pharyngitis	Nasal, Pharynx	Ocular, Oral, Skin	Ulcer, Erosion, Scar	Inflammatory infiltrates in the upper dermis with eosinophils	DIF: linear deposition of IgG/C3/IgA anti-BMZ	Prednisolone + doxycycline	Deterioration of condition (inability to swallow solid food)	/
Lagos-Villaseca, A. et al,^[21]^ 2023	84	Male	Dysphonia, Pain when swallowing	Larynx (glottis)	/	Erythema	/	/	A course of amoxicillin-clavulanate + nystatin	Deterioration of condition (dysphagia, cough, hoarseness, dyspnea)	Prednisolone + methotrexate (Significant remission)
Rousset, L. et al,^[22]^ 2023	60	Male	Dyspena, chronic dry cough	Nasal, Larynx	Ocular, Bronchial	/	Neutrophilic and eosinophilic infiltrates	DIF: linear deposition of IgG/C3 anti-BMZ ELISA/IB: anti-a6b4 integrin	Oral cyclophosphamide 2 mg/kg/d + dapsone 1 mg/kg/d	Deterioration of condition	Add dapsone, cyclophosphamide and rituximab 1 g (2 infusions 15 d apart) (Significant remission)
Syal, A. et al,^[3]^ 2023	85	Male	Dysphagia, Throat pain	Larynx, Pharynx	Esophagus	Stenosis, Yellow adherent exudate, Active inflammation	/	DIF: linear deposition of IgG/C3/IgA anti-BMZ	Rituximab + IVIG + 40mg daily prednisone, Tracheostomy	Partial remission	CO2 laser incision of the supraglottis and nasopharynx, balloon dilation, laryngeal steroid injection, brisement procedure

Anti-LM332 = anti-laminmin332, BMZ = basement membrane zone, DIF = direct immunofluorescence, ELISA = enzyme linked immunosorbent assay, IB = immunoblotting, IIF = indirect immunofluorescence, IVIG = intravenous immunoglobulin.

## 4. Discussion

Based on our findings, we present the largest case series to date concerning MMP affecting the upper respiratory tract. Both our case series and the accompanying literature review identified patients in whom upper respiratory tract symptoms were the initial manifestations of the condition. Common symptoms included rhinorrhagia, nasal obstruction, cough, hoarseness, sore throat, a sensation of a foreign body in the pharynx, dyspnea, and dysphagia. Consequently, MMP may commence with upper respiratory symptoms. These manifestations closely resemble those of several prevalent conditions, such as pharyngitis, pneumonia, asthma, vocal cord polyps, and laryngeal masses. The delay in diagnosis is frequently attributable to the nonspecific nature of these symptoms, which may lead clinicians to overlook the possibility of MMP until critical conditions, such as dyspnea, arise. As healthcare professionals, it is imperative to consider MMP when patients present with these unexplained symptoms. The clinical manifestations observed in patients are often correlated with the severity of the lesions. In our case series and literature review, the lesions exhibited included erythema, ulceration, pseudomembrane formation, and scar formation, which aligns with findings reported in the literature. In the early stages, when the lesions are small, clinicians may mistakenly perceive them as mere erythema and ulceration, leading to a reluctance to perform biopsies, which can further delay diagnosis. While erythema and ulceration typically do not elicit severe symptoms, the development of scarring that involves the upper airway can result in significant respiratory distress or even life-threatening conditions.

MMP can be diagnosed directly through DIF and pathological examination, allowing for differentiation from other diseases. MMP that affects the upper respiratory tract does not exhibit histological or immunofluorescence differences compared to MMP affecting other anatomical sites. In our study, 2 cases demonstrated linear deposits of IgG and C3 along the basement membrane, while 2 cases exhibited IgM deposits, with one patient showing a minor presence of IgA on the basement membrane. These findings align with existing literature and do not significantly differ from MMP observed at other locations.^[3,6–25]^ However, we encountered challenges with the use of laryngeal biopsy forceps, which proved to be inconvenient, and it was difficult to obtain samples that included both the mucosa and submucosa. This sampling difficulty represents a significant obstacle in the diagnostic process. ELISA and IB were conducted in 5 of the 17 cases reviewed in the literature. Among these, 4 cases yielded positive results for the presence of anti-LM-332. Furthermore, previous studies have indicated a potential association between anti-LM-332 and laryngeal involvement.^[26]^ Anti-LM-332 may correlate with more severe disease manifestations and has been implicated in the development of various malignant solid tumors, including those of the lung, breast, prostate, tongue, uterus, and bladder.^[1,2,27–29]^ Consequently, we posit that anti-LM-332 is an antibody that merits further investigation. Additionally, there is a need to identify other antibodies associated with upper respiratory tract involvement.

In the context of treatment, previous guidelines have classified cases involving multiple mucosal sites, such as the upper respiratory tract, as belonging to the “high risk” category. Localized corticosteroids typically do not prevent disease progression and scarring; therefore, early diagnosis and prompt systemic treatment utilizing a combination of corticosteroids and immunosuppressants are crucial to avert more severe complications.^[30]^ Currently, there is no consensus regarding the selection of immunosuppressants for MMP affecting the upper respiratory tract. Consequently, treatment decisions should be made comprehensively, taking into account the severity of the patient’s condition as well as the potential benefits and adverse effects associated with the immunosuppressants. Cyclophosphamide, which is linked to complications such as hemorrhagic cystitis, cancer, infertility, and severe hepatorenal toxicity,^[31]^ is best reserved for patients with refractory or severe MMP. Methotrexate is associated with adverse reactions including gastrointestinal disturbances, pancytopenia, liver dysfunction, and liver fibrosis; however, folic acid supplementation may mitigate or prevent these side effects.^[30]^ Mycophenolate mofetil is contraindicated in patients with gastrointestinal ulcers, necessitating testing for pulmonary infections prior to its use. When administering azathioprine, monitoring of thiopurine methyltransferase (TPMT) levels is essential, and attention should be given to variations in NUDT15, which may lead to more severe side effects compared to mycophenolate mofetil and methotrexate.^[32,33]^ Cyclosporine is susceptible to interactions with food and other medications, thus necessitating careful monitoring of blood concentrations, particularly when administered in high doses.^[34]^ Further clinical trials are warranted to establish the optimal pharmacological approach. In cases 1 and 2, systemic administration of glucocorticoids in conjunction with methotrexate during the early stages of the disease yielded favorable outcomes. In case 3, the patient’s condition was significantly improved through the use of intravenous immunoglobulin followed by rituximab treatment. There remains a divergence of opinions regarding the application of rituximab in mucocutaneous pemphigoid. A cohort study conducted by Lytvyn et al demonstrated that rituximab was effective in high-risk MMP patients who were unresponsive to conventional therapies.^[4]^ Additionally, a case reported by Chapman et al indicated successful therapeutic outcomes with the combination of rituximab and mycophenolate mofetil.^[2]^ It has been proposed that rituximab may require an extended duration to achieve disease control and may exhibit limited efficacy in cases of mucosal involvement attributed to IgA. Furthermore, it is recommended that rituximab be administered early in the disease progression, as its capacity to prevent scarring is contingent upon the specific mucosal site affected and the severity of the disease.^[30,35–37]^ Consequently, further comprehensive studies are warranted to evaluate the efficacy of rituximab in the context of MMP and to elucidate the underlying mechanisms involved.

The alimentary tract, which is extensively covered by mucosal tissue from the oral cavity to the anus, warrants significant attention. Following a comprehensive review of the literature, we identified that all retrieved studies focused on esophageal involvement. Clinically, esophageal involvement may present with symptoms such as dysphagia, odynophagia, oral or throat pain, weight loss, and/or an inability to consume solid food. These symptoms may overlap with those associated with laryngeal and pharyngeal involvement.^[38]^ Consequently, gastrointestinal endoscopy should be conducted to rule out esophageal involvement in patients exhibiting these symptoms. Endoscopic findings consistent with MMP may include erythema, erosion, ulceration, scarring, and stenosis.^[39]^ Notably, the esophagus may also exhibit a desquamation phenomenon.^[39]^ For cases of MMP affecting the esophagus, DIF and histopathological examination remain the gold standards for diagnosis, and these can be complemented by ELISA and IB. The findings align with those observed in MMP affecting other anatomical sites. In the cases documented by Benoit et al^[39]^ patients exhibiting both IgA and IgG antibodies demonstrated more severe symptoms, a trend that was also evident in our case series and literature review. MMP involving the esophagus should be classified as a high-risk condition, necessitating a treatment regimen that combines corticosteroids and immunosuppressants.

Given the extensive scope of MMP, it is essential to adopt a multidisciplinary approach that integrates the diagnosis and treatment across dermatology, otolaryngology, ophthalmology, gastroenterology, and other relevant fields.

## 5. Conclusion

This case series and literature review indicate that MMP should be considered in patients presenting with unexplained upper respiratory symptoms. It is essential to evaluate the extent of the disease using an electronic rhinolaryngoscope and to establish an early diagnosis through pathology and immunofluorescence techniques. The administration of corticosteroids in conjunction with immunosuppressants should be initiated promptly to manage the disease effectively and mitigate the risk of severe complications. Additionally, testing for anti-LM-332 antibodies is particularly significant, as it may be linked to tumors or other adverse outcomes. Furthermore, involvement of the alimentary tract warrants attention, as some patients may exhibit gastrointestinal symptoms as their sole manifestation, although this occurrence is rare.

## Author contributions

**Conceptualization:** Yuqi Wang, Jian Zou.

**Data curation:** Yuqi Wang, Xingli Zhou, Jie Yu, Yuhao Zou, Haiyang Wang, Wei Li, Jian Zou.

**Formal analysis:** Yuqi Wang, Jian Zou.

**Funding acquisition:** Jian Zou.

**Investigation:** Yuqi Wang, Jian Zou.

**Methodology:** Yuqi Wang, Xingli Zhou, Jie Yu.

**Project administration:** Yuqi Wang, Jian Zou.

**Resources:** Yuqi Wang, Jie Yu.

**Software:** Yuqi Wang, Xingli Zhou.

**Supervision:** Yuqi Wang, Jie Yu.

**Validation:** Yuqi Wang, Xingli Zhou.

**Visualization:** Yuqi Wang, Xingli Zhou, Jie Yu.

**Writing—original draft:** Yuqi Wang, Yuhao Zou.

**Writing—review & editing:** Haiyang Wang, Wei Li, Jian Zou.
